# Supplementary material for: Aldosterone synthase inhibitors in uncontrolled and resistant hypertension: A phenotype-stratified systematic review and network meta-analysis of randomized trials
Source: PLoS One. 2026 Jun 3;21(6):e0349932. doi: 10.1371/journal.pone.0349932 (PMC13232938; doi:10.1371/journal.pone.0349932)
Supplement: S1 File — (PDF) [file pone.0349932.s001.pdf]

NIHR | National Institute for Health and Care Research

PROSPERO
International prospective register of systematic reviews

Comparative Efficacy and Safety of Aldosterone Synthase Inhibitors (Baxdrostat and Lorundrostat) in Uncontrolled and Treatment-Resistant Hypertension: An Updated Network Meta-analysis of Randomized Controlled Trials

Ismaila Yusuf, Micah Okwah

Citation
Ismaila Yusuf, Micah Okwah. Comparative Efficacy and Safety of Aldosterone Synthase Inhibitors (Baxdrostat and Lorundrostat) in Uncontrolled and Treatment-Resistant Hypertension: An Updated Network Meta-analysis of Randomized Controlled Trials. PROSPERO 2025 CRD420251266257. Available from https://www.crd.york.ac.uk/PROSPERO/view/CRD420251266257.

REVIEW TITLE AND BASIC DETAILS

Review title

Comparative Efficacy and Safety of Aldosterone Synthase Inhibitors (Baxdrostat and Lorundrostat) in Uncontrolled and Treatment-Resistant Hypertension: An Updated Network Meta-analysis of Randomized Controlled Trials

Condition or domain being studied

Essential Hypertension; Aldosterone; Renin-angiotensin-system acting agents; Hypertensive disorder

Uncontrolled hypertension and treatment-resistant hypertension in adults.

Rationale for the review

Uncontrolled and treatment-resistant hypertension remain major causes of cardiovascular morbidity and mortality, in part due to aldosterone dysregulation. Selective aldosterone synthase inhibitors, including baxdrostat and lorundrostat, represent a novel therapeutic approach and have shown clinically meaningful blood pressure reductions in recent randomized controlled trials, including phase 3 studies and trials.

To date, no head-to-head trials comparing these agents exist, and prior systematic reviews and network meta-analyses were largely based on early-phase data. The availability of newly published randomized evidence warrants an updated network meta-analysis to indirectly compare the efficacy and safety of baxdrostat and lorundrostat using placebo as a common comparator, thereby providing contemporary evidence to inform clinical decision-making in patients with difficult-to-control hypertension.

Review objectives

Among adults with uncontrolled or treatment-resistant hypertension, what are the comparative effects of aldosterone synthase inhibitors (baxdrostat and lorundrostat) on blood pressure reduction and safety outcomes, based on randomized controlled trials?

Keywords

Comparative efficacy; Safety; Baxdrostat; lorundrostat; Uncontrolled hypertension; Treatment resistant hypertension; Randomized controlled trials; Network meta-analysis

Country

United States of America; Nigeria; Pakistan

ELIGIBILITY CRITERIA

Population

Included
Adults (≥18 years) with:

- Uncontrolled hypertension (blood pressure above guideline-recommended targets despite ≥2 antihypertensive agents), and/or

- Treatment-resistant hypertension (blood pressure uncontrolled despite ≥3 antihypertensive agents of different classes, including a diuretic).

Intervention(s) or exposure(s)

Included
The intervention is aldosterone synthase inhibitors

- Baxdrostat (any dose or dosing strategy) vs placebo

- Lorundrostat (any dose or dosing strategy) vs placebo

Comparator(s) or control(s)

Included
PICO tags selected: Placebo

Study design

Only randomized study types will be included.

Context

Aldosterone dysregulation is increasingly recognized as a central mechanism in uncontrolled and treatment-resistant hypertension. Selective aldosterone synthase inhibitors are a novel therapeutic class designed to reduce aldosterone production upstream of mineralocorticoid receptor activation. Baxdrostat and lorundrostat are the most advanced agents in this class and have been evaluated in multiple randomized controlled trials across diverse populations, including patients with chronic kidney disease.

Despite growing evidence, direct comparative trials between baxdrostat and lorundrostat are lacking, and existing systematic reviews have primarily relied on early-phase data. Recently published phase 3 trials provide new high-quality evidence that has not yet been fully synthesized. This review is conducted to place these new data in context and to provide an updated, comparative assessment of aldosterone synthase inhibitors in patients with difficult-to-control hypertension.

TIMELINE OF THE REVIEW

Date of first submission to PROSPERO
17 December 2025

Review timeline
Start date: 15 December 2025. End date: 16 January 2026.

Date of registration in PROSPERO
18 December 2025

AVAILABILITY OF FULL PROTOCOL

Availability of full protocol
A full protocol has been written but is not available because:

The full protocol is undergoing final review by the authors and will be published when ready

SEARCHING AND SCREENING

Search for unpublished studies
Both published and unpublished studies will be sought.

Main bibliographic databases that will be searched
The main databases to be searched are *CENTRAL - Cochrane Central Register of Controlled Trials*, *Embase - Embase via Ovid*, *Embase.com*, *MEDLINE*, *PubMed* and *Scopus*.

Search language restrictions
The review will only include studies published in English.

Other methods of identifying studies

Other studies will be identified by: *contacting authors or experts*, *looking through all the articles that cite the papers included in the review ("snowballing" or forward citation searching)*, *reference list checking (backward citation searching)*, *searching conference proceedings*, *searching dissertation and thesis databases* and *searching trial or study registers*.

Link to search strategy

A full search strategy has been uploaded to PROSPERO. The PDF may be accessed through this link https://www.crd.york.ac.uk/PROSPEROFILES/e9826422e699c1fe787084cd0c623020.pdf.

Selection process

Studies will be screened independently by at least two people (or person/machine combination) with a process to resolve differences.

DATA COLLECTION PROCESS

Data extraction from published articles and reports

Data will be extracted independently by at least two people (or person/machine combination) with a process to resolve differences.

Authors will be asked to provide any required data not available in published reports.

Study datasets/IPD will be obtained from study investigators or via a data repository

Study risk of bias or quality assessment

Risk of bias will be assessed using: *Cochrane RoB-2*

Data will be assessed independently by at least two people (or person/machine combination) with a process to resolve differences.

Additional information will be sought from study investigators if required information is unclear or unavailable in the study publications/reports.

Reporting bias assessment

Risk of bias due to missing results will be assessed

Certainty assessment

Certainty of evidence will be assessed using GRADE protocol

OUTCOMES TO BE ANALYSED

Main outcomes

Change in seated (office or automated office) systolic blood pressure (SBP) from baseline to the primary trial endpoint

Additional outcomes

- Change in diastolic blood pressure (DBP)

- Change in 24-hour ambulatory systolic blood pressure (when reported)

- Incidence of hyperkalemia

- Renal adverse events (e.g., decline in estimated glomerular filtration rate)

PLANNED DATA SYNTHESIS

Strategy for data synthesis

A Bayesian or frequentist network meta-analysis will be conducted using a random-effects model. The network will be placebo-anchored, allowing indirect comparison between baxdrostat and lorundrostat. Effect estimates will be reported as mean differences (MDs) with 95% confidence or credible intervals.

CURRENT REVIEW STAGE

Stage of the review at this submission

Review stage	Started	Completed
Pilot work		
Formal searching/study identification		
Screening search results against inclusion criteria		
Data extraction or receipt of IPD		
Risk of bias/quality assessment		
Data synthesis		

Review status

The review is currently planned or ongoing.

Publication of review results

Results of the review will be published.

REVIEW AFFILIATION, FUNDING AND PEER REVIEW

Review team members

Dr Ismaila Yusuf (review guarantor and contact) Guthrie Robert Packer Hospital. United States of America.

No conflict of interest declared.

Dr Micah Okwah. Yale University. United States of America.

No conflict of interest declared.

Named contact

Dr Ismaila Yusuf (ismailajayi01@gmail.com). Guthrie Robert Packer Hospital. United States of America.

Review affiliation

Guthrie Robert Packer Hospital, USA

Yale University, USA

Funding source

Review has no funding and no agreed support from an academic institution and is done in authors' own time.

Peer review

There has been no peer review of this planned review.

ADDITIONAL INFORMATION

Review conflict of interest

Declared individual interests are recorded under team member details.. No additional interests are recorded for this review.

Medical Subject Headings

Adult; Blood Pressure; Cytochrome P-450 CYP11B2; Humans; Hypertension; Network Meta-Analysis; Randomized Controlled Trials as Topic

SIMILAR REVIEWS

Check for similar records already in PROSPERO

PROSPERO identified a number of existing PROSPERO records that were similar to this one (last check made on 15 December 2025). These are shown below along with the reasons given by that the review team for the reviews being different and/or proceeding.

- Clinical Outcomes of Novel Aldosterone Synthase Inhibitors (Lorundrostat and Baxdrostat) in Uncontrolled Hypertension: A Systematic Review and Meta-Analysis of Randomized Controlled Trials [published 16 October 2025] [CRD420251170079]. The review was acknowledged as **similar** but the authors opted to continue because *the review will be more up to date*, *there are differences in intervention or comparator*, *the review uses improved methods*
- Aldosterone Synthase Inhibitors for Resistant or Uncontrolled Hypertension: A Network Meta-Analysis of Randomized Clinical Trials [published 9 October 2025] [CRD420251165200]. The review was acknowledged as **similar** but the authors opted to continue because *the review will be more up to date*
- Comparative Efficacy and Safety of Aldosterone Synthase Inhibitors in Hypertension: A Bayesian Network Meta-Analysis of Randomized Controlled Trials [published 9 April 2025] [CRD420251024035]. The review was acknowledged as **similar** but the authors opted to continue because *the review will be more up to date*

PROSPERO version history

- Version 1.0, published 18 Dec 2025

Disclaimer

The content of this record displays the information provided by the review team. PROSPERO does not peer review registration records or endorse their content.

PROSPERO accepts and posts the information provided in good faith; responsibility for record content rests with the review team. The guarantor for this record has affirmed that the information provided is truthful and that they understand that deliberate provision of inaccurate information may be construed as scientific misconduct.

PROSPERO does not accept any liability for the content provided in this record or for its use. Readers use the information provided in this record at their own risk.

Any enquiries about the record should be referred to the named review contact
