# Supplementary material for: Aldosterone synthase inhibitors in uncontrolled and resistant hypertension: A phenotype-stratified systematic review and network meta-analysis of randomized trials
Source: PLoS One. 2026 Jun 3;21(6):e0349932. doi: 10.1371/journal.pone.0349932 (PMC13232938; doi:10.1371/journal.pone.0349932)
Supplement: S2 File — (DOCX) [file pone.0349932.s002.docx]

**Comparative effectiveness of aldosterone synthase inhibitors in uncontrolled and resistant hypertension:**

*A phenotype-based network and pairwise Meta-analysis of Randomized Trials*

**Search window:** Database inception to January 14, 2026

**Last search date:** January 14, 2026

**Study design filter:** Randomized controlled trials (RCTs); human studies

# Databases Searched

- PubMed/MEDLINE
- Embase
- Cochrane Central Register of Controlled Trials (CENTRAL)
- Scopus
- ClinicalTrials.gov (results and registry cross-check)
- Backward citation searching of relevant reviews and included trials

# Sample Search Strategy: PubMed/MEDLINE

The PubMed strategy combined intervention terms (aldosterone synthase inhibition and specific agents) with hypertension phenotype terms and an RCT filter. Animal-only studies were excluded.

**Full PubMed query:**

(
 "Aldosterone Synthase Inhibitor*" OR
 baxdrostat OR
 lorundrostat OR
 osilodrostat OR
 LCI699 OR
 "CYP11B2 inhibitor*"
)
AND
(
 "Hypertension"[MeSH] OR
 hypertension OR
 "resistant hypertension" OR
 "treatment resistant hypertension" OR
 "uncontrolled hypertension"
)
AND
(
 randomized controlled trial[pt] OR
 controlled clinical trial[pt] OR
 randomized[tiab] OR
 randomised[tiab] OR
 randomly[tiab] OR
 placebo[tiab] OR
 trial[ti]
)
NOT
(
 animals[mh] NOT humans[mh]
)

# Adaptation to Other Databases

The PubMed strategy was adapted to other databases using database-specific controlled vocabulary (e.g., Emtree in Embase) and syntax. Equivalent field restrictions (title/abstract keywords), RCT filters, and human-only limits were applied where available.

# Key Concepts and Synonyms

| Concept | Representative terms |
| --- | --- |
| Intervention | Aldosterone synthase inhibitor; CYP11B2 inhibitor; baxdrostat; lorundrostat; osilodrostat; LCI699 |
| Condition | Hypertension; resistant hypertension; treatment resistant hypertension; uncontrolled hypertension |
| Study design | Randomized controlled trial; controlled clinical trial; randomized; placebo; trial |

# Reproducibility Notes

- Searches were run from inception through January 14, 2026.
- Conference abstracts and unpublished trials were assessed via ClinicalTrials.gov and citation searching.
- The final set of included studies comprised 7 randomized controlled trials.
