# Supplementary material for: Aldosterone synthase inhibitors in uncontrolled and resistant hypertension: A phenotype-stratified systematic review and network meta-analysis of randomized trials
Source: PLoS One. 2026 Jun 3;21(6):e0349932. doi: 10.1371/journal.pone.0349932 (PMC13232938; doi:10.1371/journal.pone.0349932)
Supplement: S3 File — (PDF) [file pone.0349932.s003.pdf]

League tables for all network meta-analysis

1. Systolic blood pressure

Drug	P-score (common)	P-score (random)
Lorundrostat	0.9948	0.8985
Baxdrostat	0.623	0.6792
LCI699	0.3821	0.422
Placebo	0.0001	0.0003

2. Diastolic blood pressure

Drug	P-score (common)	P-score (random)
Lorundrostat	0.6484	0.8108
Baxdrostat	0.8516	0.6891
Placebo	0.00	0.00

3. Resistant hypertension

Drug	P-score (common)	P-score (random)
Lorundrostat	0.8242	0.8342
Baxdrostat	0.8341	0.8189
LCI699	0.3345	0.3357
Placebo	0.0072	0.0113

4. Uncontrolled hypertension

Drug	P-score (common)	P-score (random)
LCI699	0.9365	0.9365
Lorundrostat	0.5634	0.5634
Placebo	0.0001	0.0001
