# Supplementary material for: Aldosterone synthase inhibitors in uncontrolled and resistant hypertension: A phenotype-stratified systematic review and network meta-analysis of randomized trials
Source: PLoS One. 2026 Jun 3;21(6):e0349932. doi: 10.1371/journal.pone.0349932 (PMC13232938; doi:10.1371/journal.pone.0349932)

Discontinuation due to adverse events

1. Forest plot

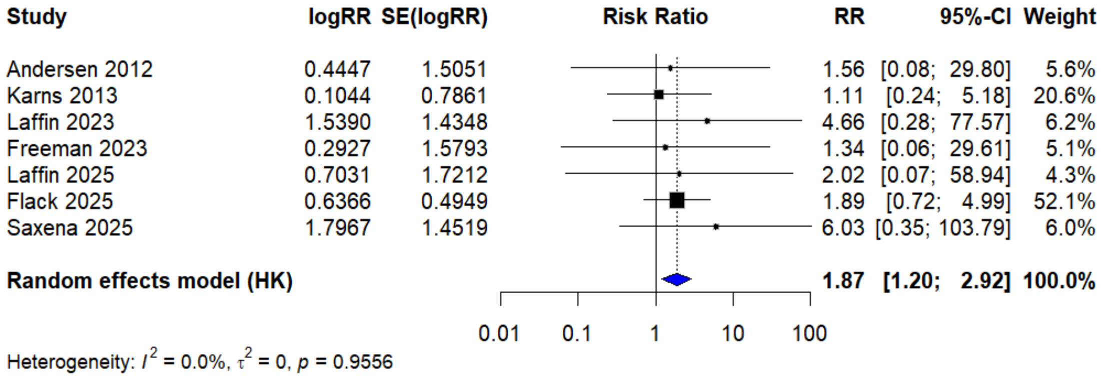

2. Funnel Plot

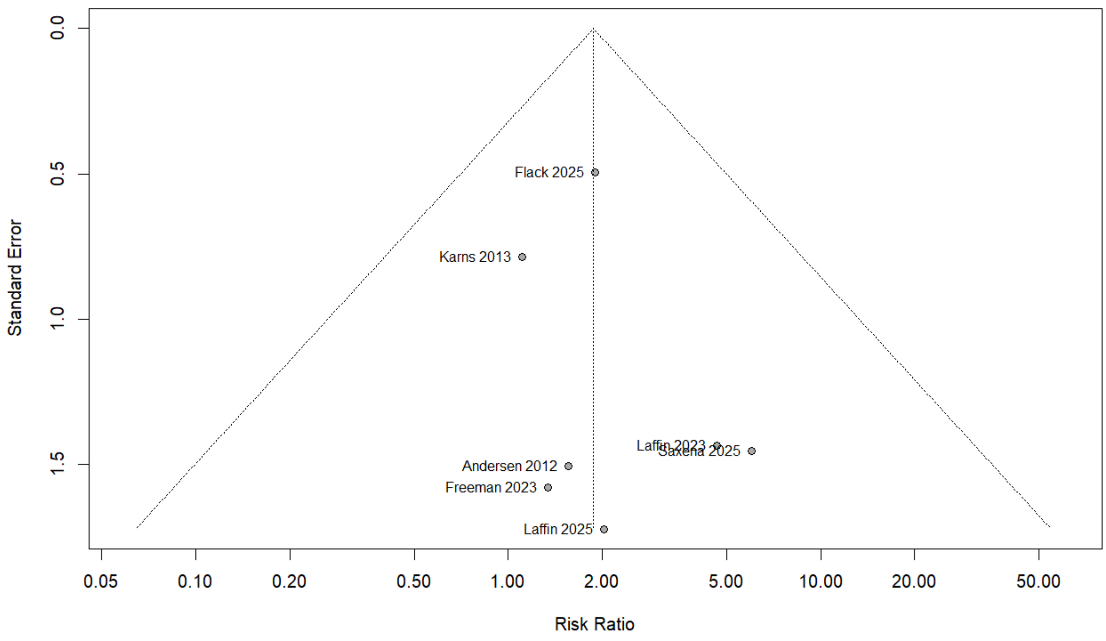

3. Leave-one-out sensitivity analyses

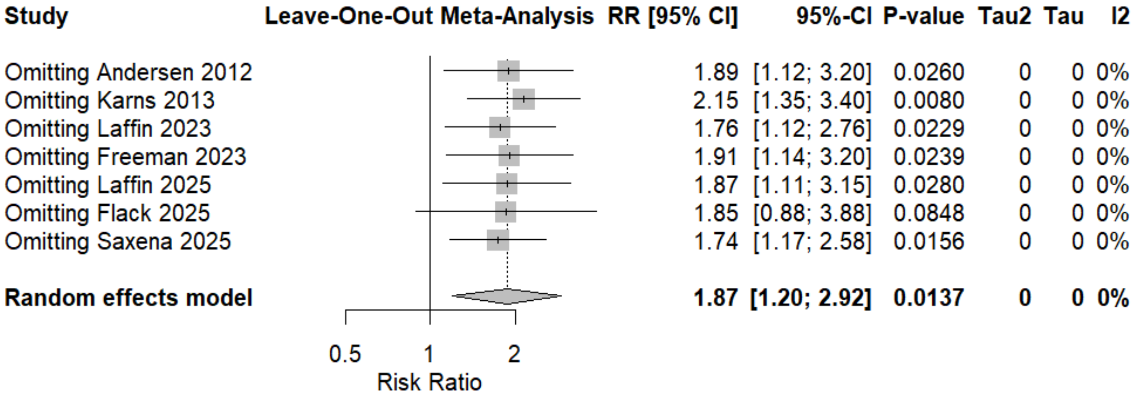

Hyperkalemia

1. Forest plot

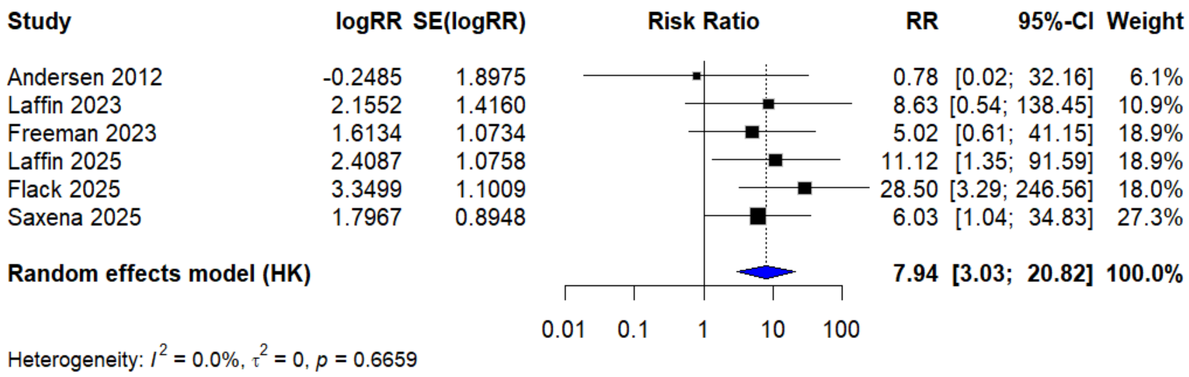

2. Funnel Plot

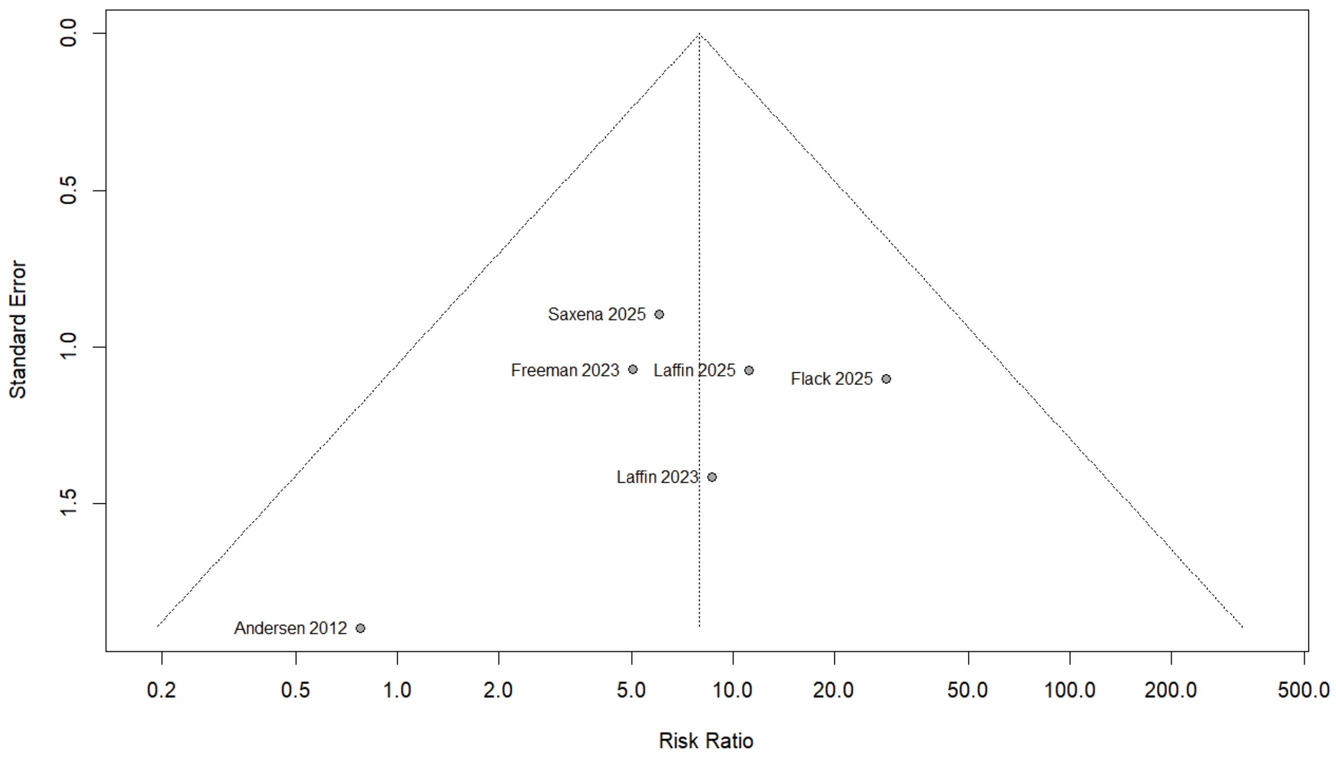

3. Leave-one-out sensitivity analyses

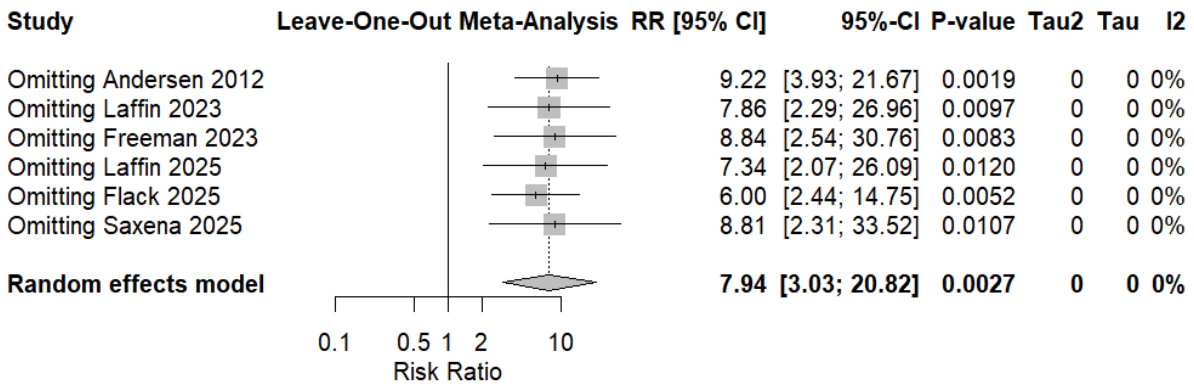

Hyponatremia

1. Forest plot

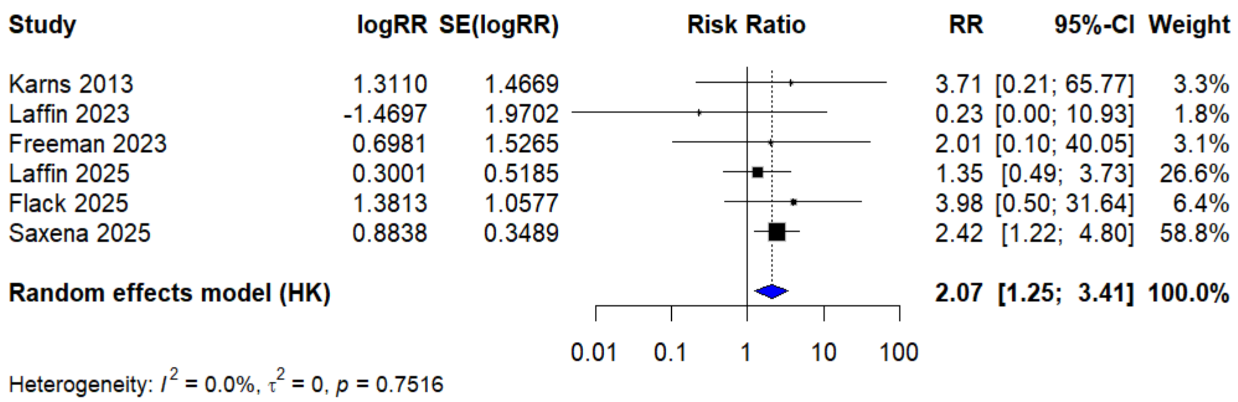

2. Funnel Plot

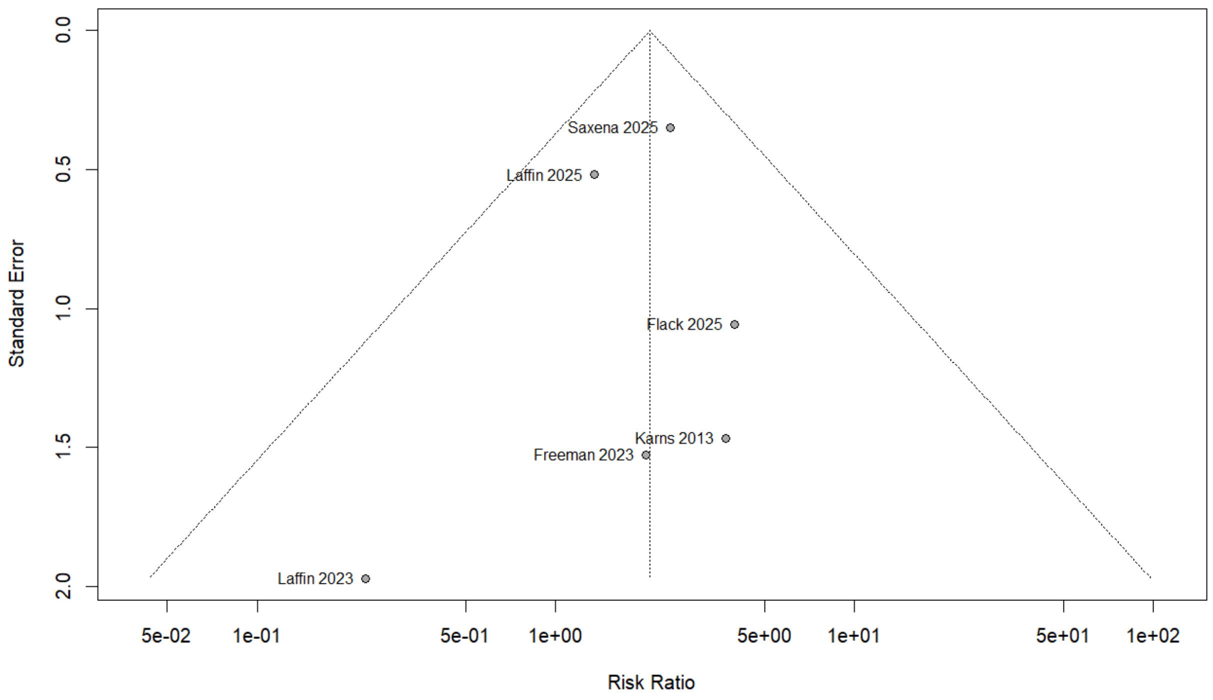

3. Leave-one-out sensitivity analyses

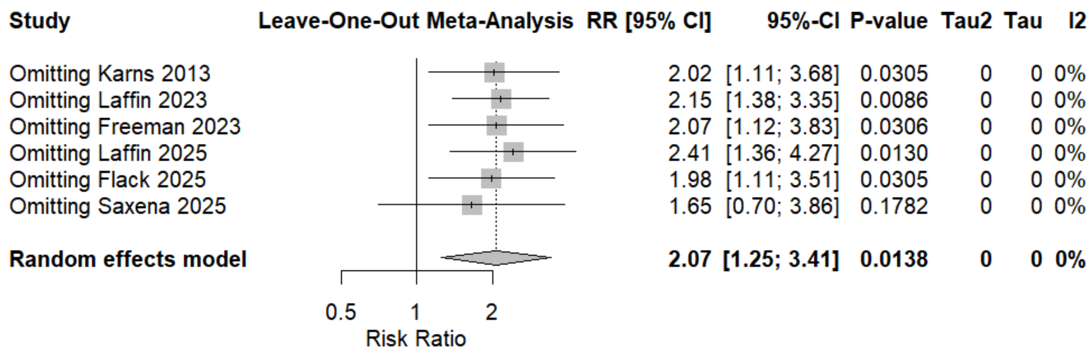

Hypotension

Study	logRR	SE(logRR)	RR	95%-CI	Weight
Laffin 2023	0.1570	1.5252	1.17	[0.06; 23.25]	7.5%
Freeman 2023	0.0000	1.6350	1.00	[0.04; 24.65]	6.5%
Laffin 2025	0.9895	0.6593	2.69	[0.74; 9.79]	40.0%
Flack 2025	1.0080	0.7663	2.74	[0.61; 12.30]	29.6%
Saxena 2025	1.6808	1.0274	5.37	[0.72; 40.22]	16.5%
**Random effects model (HK)**			**2.67**	**[1.45; 4.92]**	**100.0%**

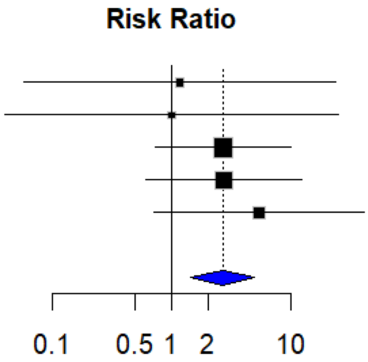

Heterogeneity: $I^2 = 0.0\%$, $\tau^2 = 0$, $p = 0.8915$

2. Funnel Plot

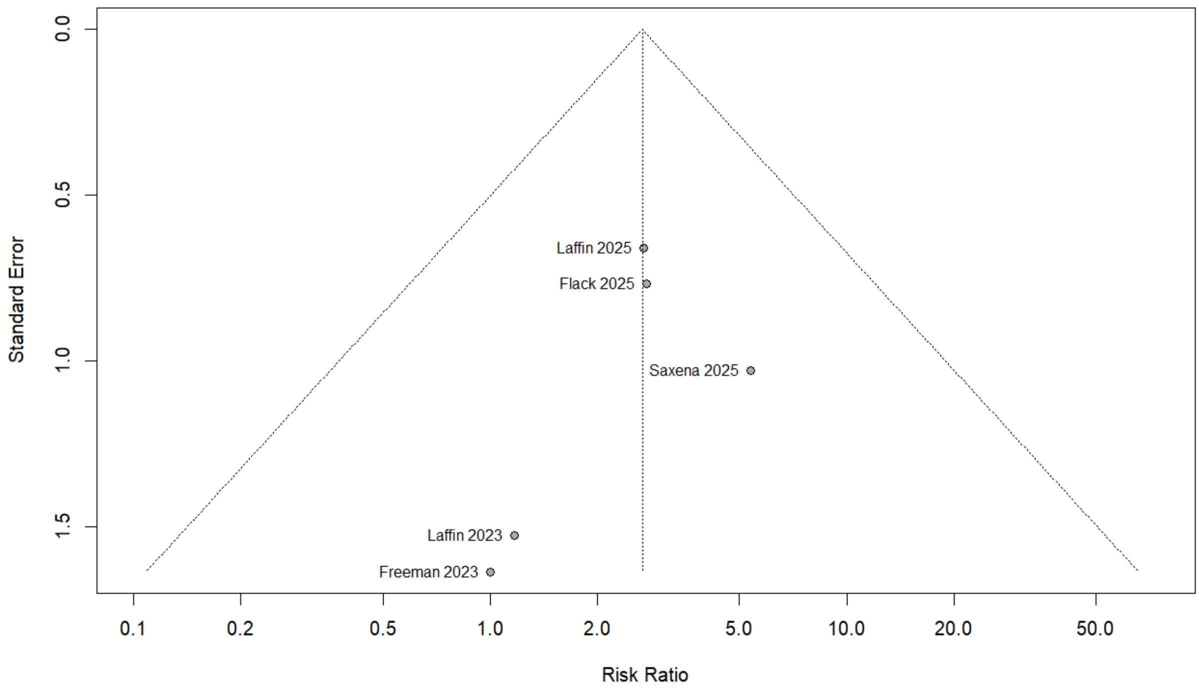

3. Leave-one-out sensitivity analyses

Study	RR [95% CI]	95%-CI	P-value	Tau2	Tau	I2
Omitting Laffin 2023	2.85	[1.40; 5.82]	0.0184	0	0	0%
Omitting Freeman 2023	2.86	[1.45; 5.63]	0.0159	0	0	0%
Omitting Laffin 2025	2.66	[0.93; 7.56]	0.0587	0	0	0%
Omitting Flack 2025	2.64	[1.01; 6.93]	0.0491	0	0	0%
Omitting Saxena 2025	2.33	[1.24; 4.37]	0.0236	0	0	0%
**Random effects model**	**2.67**	**[1.45; 4.92]**	**0.0112**	**0**	**0**	**0%**

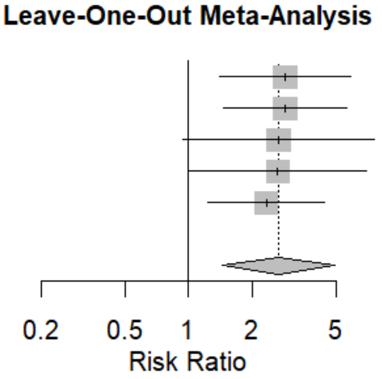

Supplement: S4 File — (PDF) [file pone.0349932.s004.pdf]
